# Histological and diffusion-weighted magnetic resonance imaging data from normal and degenerated optic nerve and chiasm of the rat

**DOI:** 10.1016/j.dib.2019.104399

**Published:** 2019-08-15

**Authors:** Omar Narvaez-Delgado, Gilberto Rojas-Vite, Ricardo Coronado-Leija, Alonso Ramírez-Manzanares, José Luis Marroquín, Ramsés Noguez-Imm, Marcos L. Aranda, Benoit Scherrer, Jorge Larriva-Sahd, Luis Concha

**Affiliations:** a Institute of Neurobiology, Universidad Nacional Autónoma de México, Blvd. Juriquilla 3001, Querétaro, Querétaro, Mexico; b Centro de Investigación en Matemáticas, Valenciana S/N, Guanajuato, Guanajuato, Mexico; c Department of Human Biochemistry, School of Medicine, University of Buenos Aires/CEFyBO, CONICET, Buenos Aires, Argentina; d Computational Radiology Laboratory, Boston Children's Hospital, Harvard Medical School, Boston, MA, USA

**Keywords:** Diffusion, Magnetic resonance imaging, Axonal degeneration, Crossing fibers, Retinal ischemia, Microstructure

## Abstract

Diffusion-weighted magnetic resonance imaging (dMRI) is widely used to infer microstructural characteristics of tissue, particularly in cerebral white matter. Histological validation of the metrics derived from dMRI methods are needed to fully characterize their ability to capture biologically-relevant histological features non-invasively. The data described here were used to correlate metrics derived from dMRI and quantitative histology in an animal model of axonal degeneration (“Histological validation of per-bundle water diffusion metrics within a region of fiber crossing following axonal degeneration” [1]). Unilateral retinal ischemia/reperfusion was induced in 10 rats, by the elevation of pressure of the anterior chamber of the eye for 90 min. Five rats were used as controls. After five weeks, injured animals were intracardially perfused to analyze the optic nerves and chiasm with dMRI and histology. This resulted in 15 brain scans, each with 80 diffusion-sensitizing gradient directions with b = 2000 and 2500 s/mm^2^ and 20 non-diffusion-weighted images (b = 0 s/mm^2^), with isometric voxel resolution of 125 μm^3^. Histological sections were obtained after dMRI. Optical microscopy photomicrographs of the optic nerves (stained with toluidine blue) are available, as well as their corresponding automatic segmentations of axons and myelin.

**Table:** Specification table

Subject	Radiology and Imaging
Specific subject area	Microstructure analysis with diffusion-weighted magnetic resonance imaging and histology
Type of data	Files
How data were acquired	1. *Ex vivo* dMRI were obtained with a 7 Tesla pre-clinical scanner. dMRI were preprocessed with MRtrix3, ANTs and FSL 5.0 software. 2. Photomicrograph mosaics of whole optic nerve sections were obtained at 63X magnification. 3. Quantitative evaluation of the photomicrograph mosaics was performed with AxonSeg 3.0. 4. Synthetic data.
Data format	* All raw photomicrographs are in.TIF format. Files derived from automatic segmentation are in.png format, and tables with individual axon information in.csv. * *Ex vivo* dMRI in .mif format (https://mrtrix.readthedocs.io/en/latest/getting_started/image_data.html) * Synthetic dMRI data in NIFTI format.
Parameters for data collection	*Ex vivo* dMRI: isometric resolution of 125 μm. 80 DWI directions, each with b = 2000 and 2500 s/mm^2^. Histology: Mosaics acquired at 63X magnification.
Description of data collection	Animals were perfused intracardially 5 weeks after unilateral retinal ischemia (n = 10). Five control animals were also analyzed. Brains were extracted and *ex vivo* dMRI were acquired. After dMRI scanning, brain specimens were processed for histology.
Data source location	National Laboratory for magnetic resonance imaging. Institute of Neurobiology, Universidad Nacional Autónoma de México, Juriquilla, Querétaro, Mexico
Data accessibility	Repository name: White Matter Microscopy Database [2]. Open Science Framework Data identification number: doi 10.17605/OSF.IO/YP4QG Direct URL to experimental data: https://osf.io/yp4qg/Direct URL to synthetic data:https://doi.org/10.5281/zenodo.3238625
Related research article	G. Rojas-Vite, R. Coronado-Leija, O. Narvaez-Delgado, A. Ramírez-Manzanares, J.L. Marroquín, R. Noguez-Imm, M.L. Aranda, B. Scherrer, J. Larriva-Sahd, L. Concha, Histological validation of per-bundle water diffusion metrics within a region of fiber crossing following axonal degeneration, NeuroImage. 201 (2019) 116013. doi:10.1016/j.neuroimage.2019.116013.

**Table:** 

**Value of the data** • These data provide a direct link between dMRI and histological features. • The data can be used as a gold-standard for validation of dMRI methods. • Histological data can also be used to construct synthetic substrates with realistic microstructure for simulations of normal and degenerated tissue.

## 1 Data

**Fig. 1:**
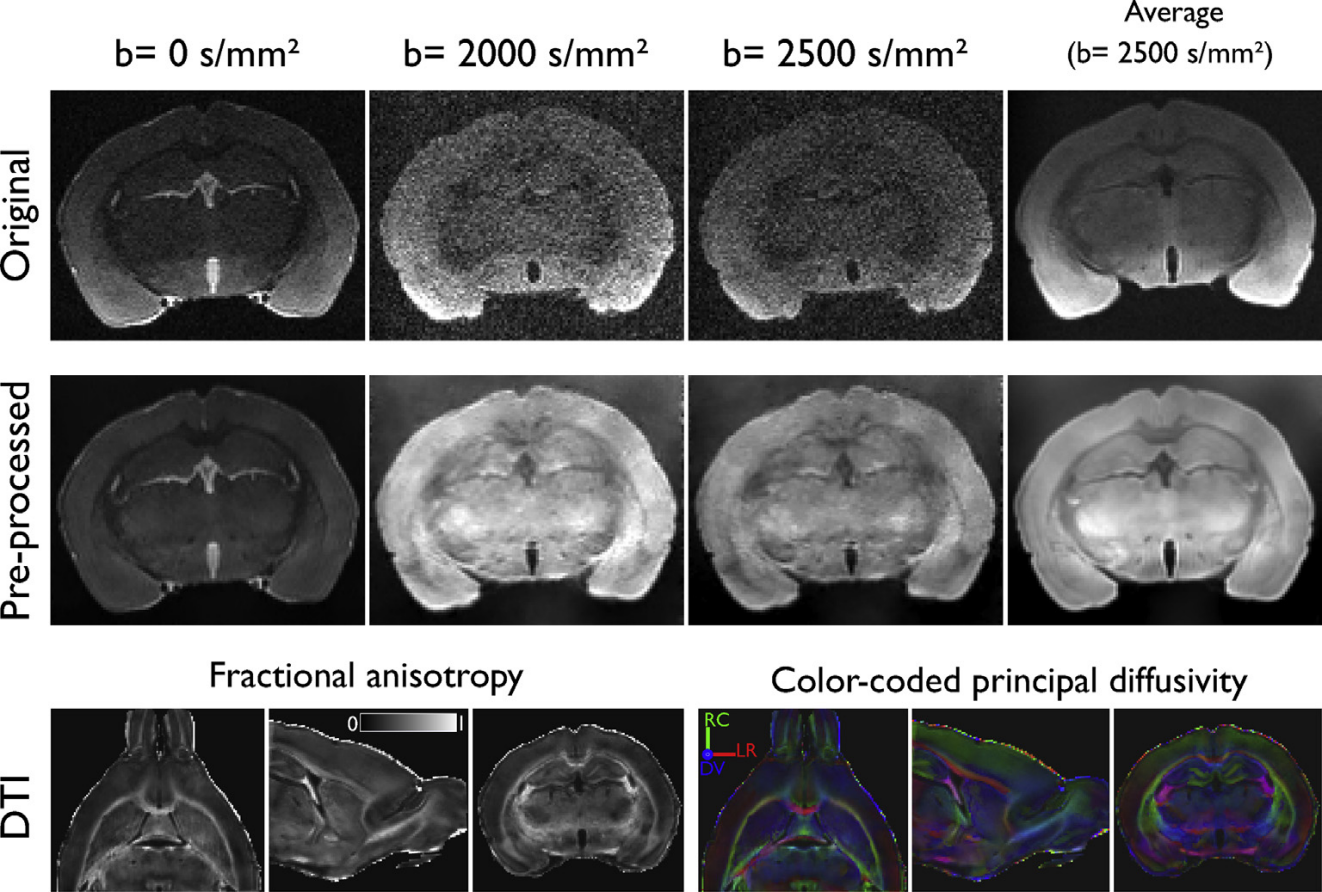
Example *ex vivo* DWI images, before and after pre-processing, and derived diffusion tensor imaging (DTI) maps. RC: rostro-caudal; LR: left-right; DV: dorso-ventral.

**Fig. 2:**
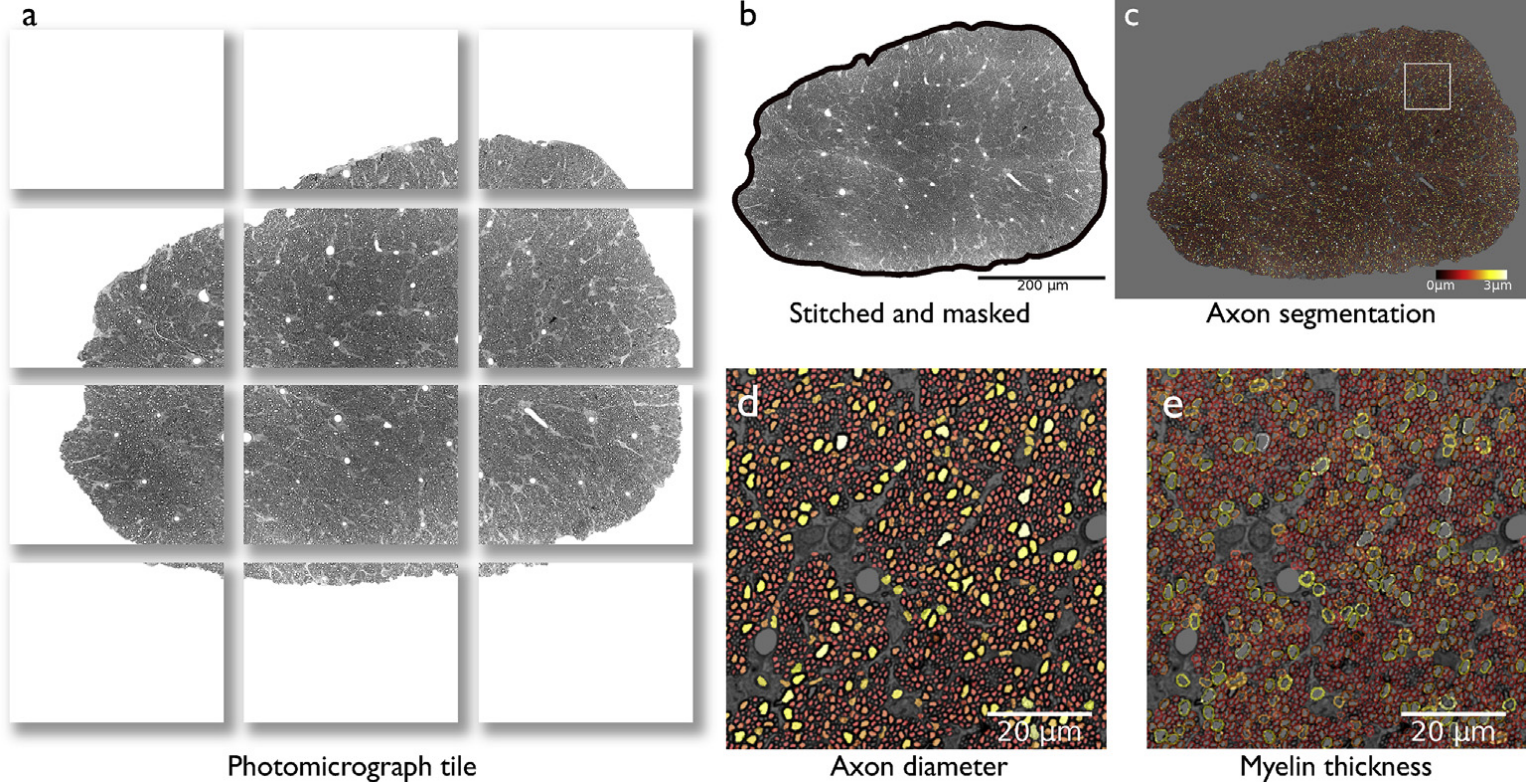
Quantitative histology. Individual microphotographs of the optic nerves (a) were digitally stitched (b), and the outline of the optic nerves were manually drawn to create a mask of the structure, within which automatic axon segmentation was performed (c). The squared region in (c) is shown enlarged in panels (d-e), displaying axon diameter and myelin thickness, respectively.

The repository contains data organized into two main folders, one for dMRI data and another for histology. The **mri** folder contains data from control and damaged (retinal ischemia) rat optic nerves. For each animal, the raw dMRI data is provided (Fig. 1), along with the pre-processed images (cropped, denoised, and corrected for eddy-current distortions and bias field inhomogeneities). Also included is a whole brain binary mask, and binary masks for the optic nerves and chiasm. The **histology** folder contains one folder for every control and experimental animal, each with three photomicrograph mosaics from each optic nerve (Fig. 2). For each mosaic, there are the corresponding axonal and myelin segmentations obtained through AxonSeg, and a comma-separated-value file (.csv) detailing the morphological parameters for every identified axon.

## 2 Experimental design, materials, and methods

### 2.1 Retinal ischemia/reperfusion and tissue fixation

Axonal degeneration was induced through unilateral retinal ischemia [3]. In this model, the histological features and severity of the induced damage of the visual pathway depends on the duration of ischemia and time of examination with respect to the procedure. The data described here correspond to chronic changes (five weeks following ischemia). Unilateral retinal ischemia was performed in 10 female Wistar rats (age 16–18 weeks, weight 354 ± 59 g). Five additional animals served as as controls.

Animals were anesthetized with a ketamine/xylazine (70/4 mg/ml) solution administered 0.1ml/100g I.P., then placed in a stereotaxic frame to reduce movement during the experimental procedure. A 32-gauge needle was injected unilaterally through the anterior chamber of the eye. The needle was connected via tubing to a reservoir containing saline solution, and the reservoir was elevated until pressure (measured via an in-line pressure gauge) reached 120mm/Hg; this pressure was maintained for 90 minutes. Retinal pallor confirmed lack of blood flow in the retina during the period of time in which pressure was elevated. The needle was carefully extracted and topical antibiotics were administered. After the experimental procedure the animals were allowed to recover from the anesthesia and placed in their cages with *ad libitum* access to food and water. Thirty-five days after retinal ischemia/reperfusion animals were deeply anesthetized with pentobarbital sodium and intracardially perfused with 4% paraformaldehyde (PFA) and 2.5% glutaraldehyde with gadobutrol (Gadovist, Bayer; 0.2 mM). Brains were stored in PFA 4% at 4 °C until MRI scanning.

### 2.2 MRI data acquisition

Samples were scanned using a 7 T Bruker Pharmascan 70/16 with 760 mT/m maximum gradient amplitude using a combination of a 72 mm inner-diameter circularly polarized radio-frequency coil (for transmission), and a rat head 2 × 2 surface array rat coil (receive-only). The scanner was controlled through Paravision 6.0.1. Imaging sessions were performed 24 ± 21(range 2–63) days after intracardiac perfusion. Brains were taken out of refrigeration 2 hours before imaging, placed in a plastic tube, and immersed in perfluoropolyether (Fomblin Y, Sigma-Aldrich). The specimens were oriented such that the chiasm and optic nerves were in close proximity to the receiving coil. Scanning was performed at room temperature (21 °C) with fluctuations of less than 1.5 °C (measured twice with an MRI-compatible thermometer [Small Animal Instruments, Inc., Stony Brook, NY] placed inside the scanner bore). A B0 map was obtained and used to aid automatic shimming before dMRI acquisition. Images were acquired with 125 × 125 × 125 μm^3^ voxel resolution using a 3-dimensional echo-planar acquisition with 8 segments. Repetition time (TR) was 250 ms and echo time (TE) of 21 ms; NEX = 1. Diffusion-weighted images (DWI) in 80 different directions were acquired, each with a b value of 2000 and 2500 s/mm^2^. The duration (δ) and separation (Δ) of the diffusion gradient were 3.1 and 10 ms, respectively. Odd and even echoes were averaged (double sampling). Additionally, 20 non-diffusion weighted volumes (b = 0 s/mm^2^) were obtained with identical parameters. Total scanning time was 15 hours. After scanning, specimens were returned to PFA and refrigerated until preparation for histology. Example images are shown in Fig. 1.

### 2.3 MRI data preprocessing and analysis

Obtained images were cropped to remove unnecessary space without tissue, then were denoised using random matrix theory [4], as implemented in the dwidenoise command, available in MRtrix^1^(version 3.0_RC3). Geometric distortions induced by eddy currents, as well as image drift during the long acquisition period, were corrected by registering each volume to the average b = 0 s/mm^2^ volume using a linear transformation with 12° of freedom using the flirt command available in the FSL suite [5]. Finally, bias field inhomogeneities were corrected using ANTs software with N4 algorithm (N4BiasFieldCorrection version 2.2.0, available in ANTS^2^) [6], with the bias field estimated on the average b = 0 s/mm^2^ volume, and the resulting correction factor applied to all volumes. Example images before and after pre-processing, along with quantitative maps derived from the diffusion tensor [7], are shown in Fig. 1.

**Fig. 3:**
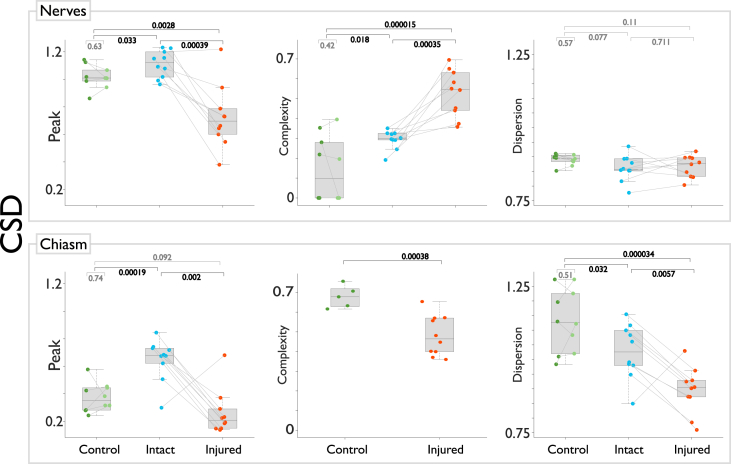
Pair-wise statistical tests for metrics derived from constrained spherical deconvolution (CSD). Paired t-tests for comparisons between left/right and injured/intact optic nerves or bundles in the chiasm. Independent samples t-tests for between-group comparisons. Peak AFD is greatly reduced in the injured nerve and corresponding axonal population in the chiasm. Complexity is normally very low in the intact nerve, but increases in the case of injury; the opposite pattern is seen in the chiasm. Dispersion is not informative in the injured optic nerve, but is reduced in the injured chiasm.

**Fig. 4:**
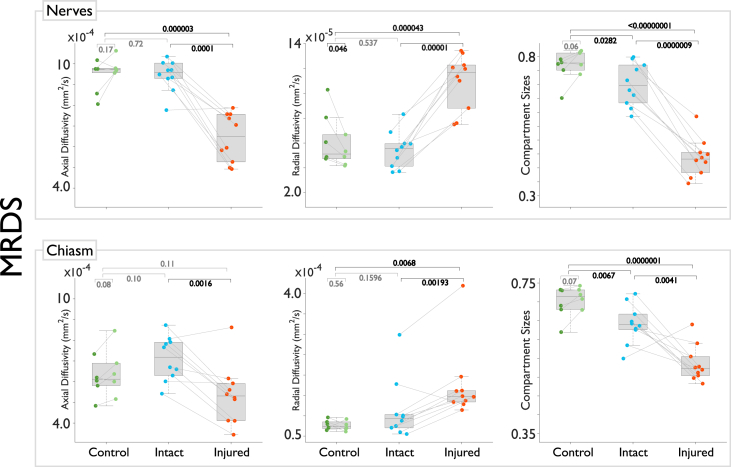
Pair-wise statistical tests for metrics derived from multi-resolution discrete search (MRDS). The injured optic nerve and corresponding bundle in the chiasm show reduced axial diffusivity, increased radial diffusivity, and reduced compartment size.

**Fig. 5:**
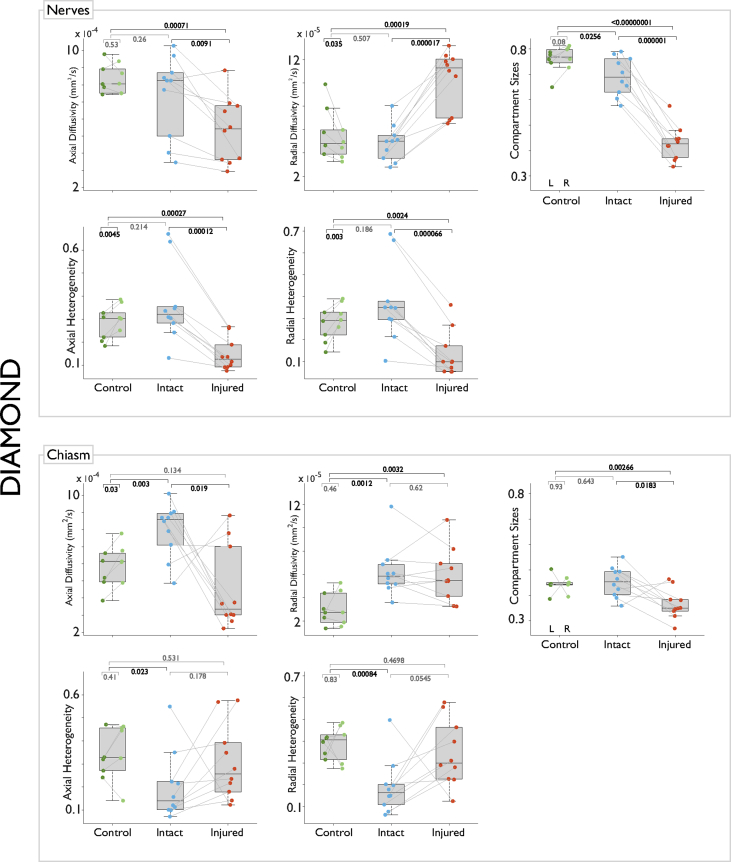
Pair-wise statistical tests for metrics derived from distribution of anisotropic microstructural environments (DIAMOND). The injured optic nerve shows reduced axial diffusivity, increased radial diffusivity, and reduced compartment size. Axial and radial heterogeneities are also reduced in injured optic nerves. In the chiasm, the injured component shows reduced axial diffusivity and compartment size, but not an increase of radial diffusivity. Axial and radial heterogeneities are abnormally low for the intact component of the chiasm, and normal for the injured part.

The dMRI data can be analyzed with a variety of diffusion models and methods. In our accompanying article we analyzed these data using three different dMRI models: constrained spherical deconvolution (CSD) [8], multi-resolution discrete search (MRDS) [9] and distribution of anisotropic microstructural environments (DIAMOND) [10]. A selection of the resulting metrics are shown in Fig. 3, Fig. 4, Fig. 5 (see our research paper for more metrics and details [1]).

### 2.4 Histological procedures

Following the dMRI acquisition, the optic nerves and chiasm were separated from the brain to proceed with histology preparation. Time between dMRI and tissue preparation was 47 ± 29 (range 11–118) days. Tissue were washed with phosphate buffer solution (0.1 M, 4 °C) three times to remove the excess of fixing solution. Staining was performed using Osmium tetroxide 1% in phosphate buffer during 1 h in rotation. After the that time, specimens were washed again with PBS. Dehydration was done in an acetone gradient (60%, 70%, 80%, 90% until absolute acetone). Then, tissue was embedded in a 2:1 epoxy resin/acetone solution for approximately 18 hours. Samples were placed in a mold with epoxy resin and heated to 60 °C for its polymerization. We obtained 500 nm thick sections of the optic nerves, oriented perpendicular to the long axis of the structures. Each section was stained with a solution of toluidine blue and sodium borate (both 0.5%).

### 2.5 Quantitative evaluation of histology

Having stained optic nerves sections, photomicrography tiles were taken with a vertical Zeiss Axio Imager microscope with motorized stage at 63 × magnification. Illumination, contrast, focus and exposure were adjusted for every frame. Tiles were stitched with the Grid/collection stitching plugin [11] available in Fiji software [12] to obtain three mosaics of 3886 × 3848 pixels of complete sections for every optic nerve (Fig. 2). Tile overlap used was 10% using linear blending as fusion method with a regression threshold of 0.30, Max/avg displacement threshold of 2.50 and absolute displacement threshold of 3.50. A total optic nerve area mask was manually outlined and used to avoid surrounding tissue that could interfere with automatic segmentation. Quantitative evaluation of the mosaics was performed with AxonSeg [13], using a single set of segmentation parameters for both control and experimental conditions (included in the data repository).

**Fig. 6:**
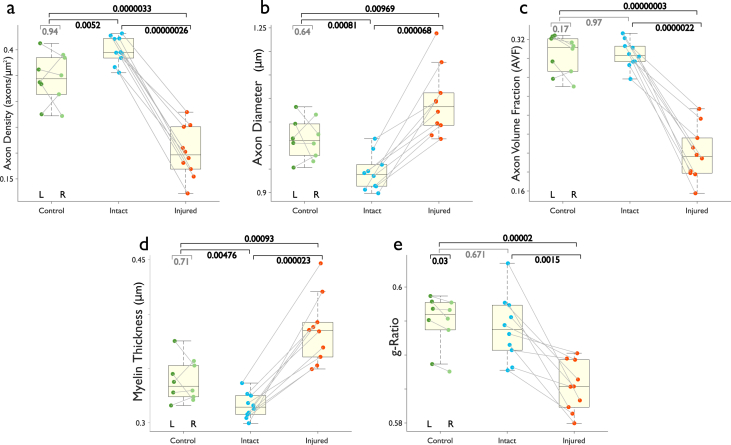
Quantitative histology of optic nerves. Each point represents the median value of each histological feature in three photomicrographs. Axonal density was greatly reduced in injured nerves following retinal ischemia. This reduction is reflected in considerable reductions of axonal and myelin volume fractions. Average axon diameter and myelin thickness increased slightly in injured nerves (thus reducing g-ratio), due to a preferential loss of small-caliber axons and an overestimation of myelin thickness caused by myelin sheath separation in injured nerves.

Metrics obtained were total axon count, axon diameter, axon + myelin diameter, axon area, myelin area, and myelin thickness, and g-ratio; from these, and total optic nerve area, we derived axon density, axon volume fraction (AVF), and myelin volume fraction (MVF). Resulting metrics are visualized in Fig. 6. We note that automatic segmentation of myelin tends to overestimate myelin thickness of degenerated axons due to fraying of myelin sheaths and intra-myelin inclusions (see accompanying article for details).

### 2.6 Synthetic data

**Fig. 7:**
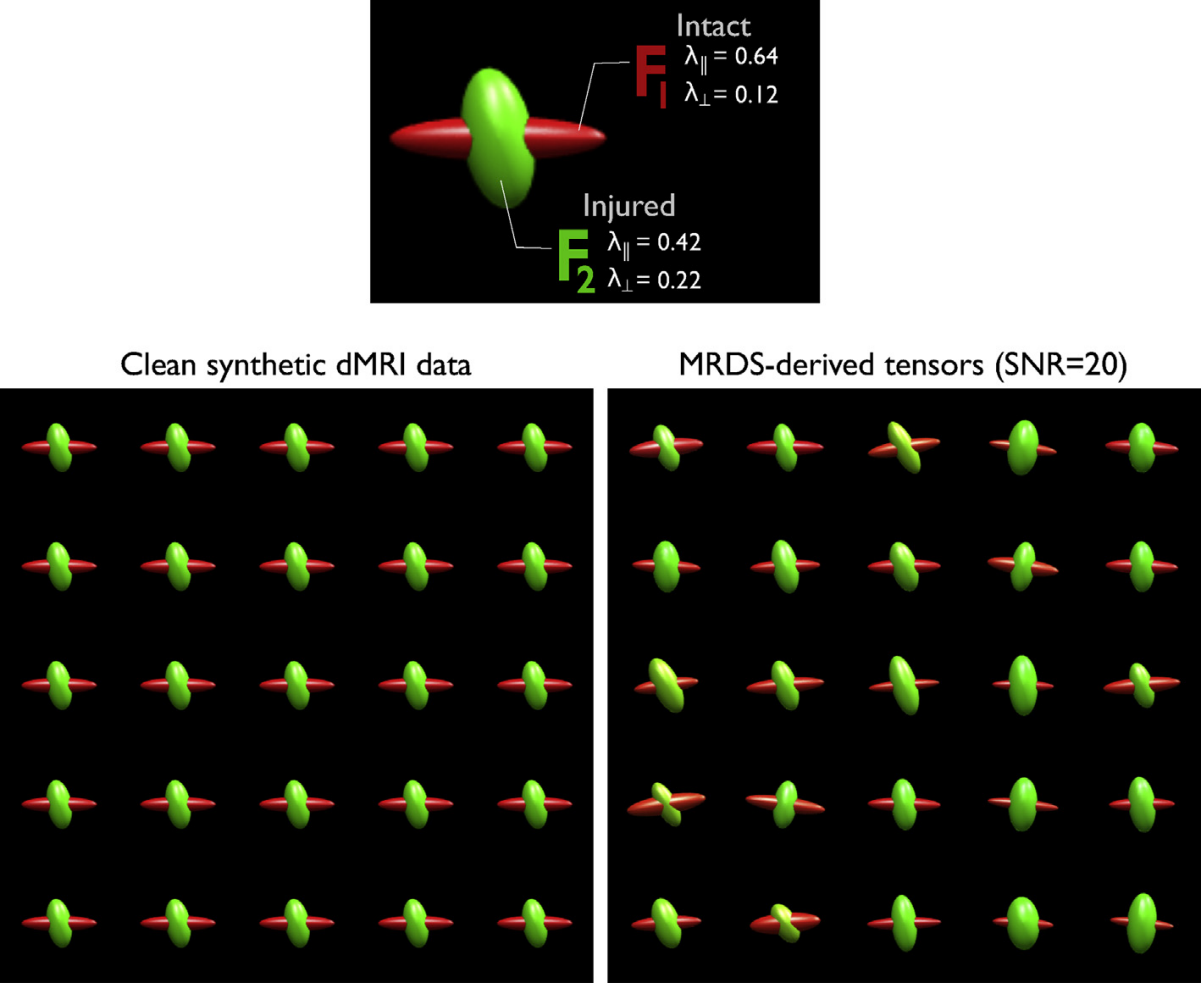
Simulated dMRI data. Two fiber populations were modelled as diffusion tensors. The tensor corresponding to intact axons (*F*_*1*_) shows high anisotropy as compared to the tensor representing injured axons (*F*_*2*_). MRDS captures the differences in anisotropy between these two synthetic axonal populations. Units for parallel and perpendicular diffusivities (λ_‖_ and λ, respectively) are in units ×10^−3^ mm^2^/s.

Analysis of dMRI of the optic chiasm using CSD revealed unexpectedly high Peak AFD of the intact bundle in the experimental animals (Fig. 3). To evaluate whether this finding is an artifact produced by CSD, we generated synthetic data to mimic the expected microstructural environment of the chiasm in the case of degeneration of only one axonal population (Fig. 7). Using the multi-tensor model, synthetic dMRI data were created consisting of 100 voxels, each with two tensors crossing at right angles. For each voxel, one tensor was modeled as an “intact” fiber bundle, following the DTI metrics observed in our experimental data (parallel diffusivity = 0.64 and perpendicular diffusivity = 0.12 × 10^−3^ mm^2^/s), while the other tensor was modeled as an “injured” fiber bundle (parallel diffusivity = 0.42 and perpendicular diffusivity = 0.22 × 10^−3^ mm^2^/s). Volume fractions were set as *f* = 0.5 for both tensors. The multi-shell scheme to generate the synthetic signals consisted of 20 *b* = 0 s/mm^2^, and 160 DWI volumes (80 diffusion gradient orientations, each with two distinct *b* values, namely *b* = 2000 and 2500 s/mm^2^). Signal-to-noise ratio was set at 20. The data set is available at https://doi.org/10.5281/zenodo.3238625.

## Footnotes

1 http://www.mrtrix.org/.

2 http://stnava.github.io/ANTs/.
